# Timing of antimicrobial prophylaxis for cesarean section is critical for gut microbiome development in term born infants

**DOI:** 10.1080/19490976.2022.2038855

**Published:** 2022-02-20

**Authors:** Verena Bossung, Mariia Lupatsii, Lkhagvademberel Dashdorj, Oronzo Tassiello, Sinje Jonassen, Julia Pagel, Martin Demmert, Ellinor Anna Wolf, Achim Rody, Silvio Waschina, Simon Graspeuntner, Jan Rupp, Christoph Härtel

**Affiliations:** a Department of Obstetrics and Gynecology, University Hospital of Schleswig-Holstein, Campus, Lübeck, Germany; b Department of Infectious Diseases and Microbiology, University of Lübeck, Lübeck, Germany; c German Center for Infection Research (DZIF), Partner Site Hamburg-Lübeck-Borstel-Riems, Lübeck, Germany; d Department of Pediatrics, University Hospital of Schleswig-Holstein, Campus, Lübeck, Germany; e Institute for Human Nutrition and Food Science, Nutriinformatics, University of Kiel, Kiel, Germany; f Department of Pediatrics, University Hospital of Würzburg, Wurzburg, Germany

**Keywords:** Microbiome, cesarean section, antibiotics, surgical antimicrobial prophylaxis, term infant, diversity, antibiotic resistome

## Abstract

Animal models imply that the perinatal exposure to antibiotics has a substantial impact on microbiome establishment of the offspring. We aimed to evaluate the effect of timing of antimicrobial prophylaxis for cesarean section before versus after cord clamping on gut microbiome composition of term born infants. We performed an exploratory, single center randomized controlled clinical trial. We included forty pregnant women with elective cesarean section at term. The intervention group received single dose intravenous cefuroxime after cord clamping (n = 19), the control group single dose intravenous cefuroxime 30 minutes before skin incision (n = 21). The primary endpoint was microbiome signature of infants and metabolic prediction in the first days of life as determined in meconium samples by 16S rRNA gene sequencing. Secondary endpoints were microbiome composition at one month and 1 year of life. In meconium samples of the intervention group, the genus Staphylococcus pre-dominated. In the control group, the placental cross-over of cefuroxime was confirmed in cord blood. A higher amino acid and nitrogen metabolism as well as increased abundance of the genera Cutibacterium, Corynebacterium and Streptophyta were noted (indicator families: Cytophagaceae, Lactobacilaceae, Oxalobacteraceae). Predictive models of metabolic function revealed higher 2ʹfucosyllactose utilization in control group samples. In the follow-up visits, a higher abundance of the genus Clostridium was evident in the intervention group. Our exploratory randomized controlled trial suggests that timing of antimicrobial prophylaxis is critical for early microbiome engraftment but not antimicrobial resistance emergence in term born infants.

## Introduction

An increasing number of infants is born by cesarean section (CS) worldwide accounting for 21.1% of all births with large variations between countries and continents.^1^ In Germany, the CS rate was 31,66% in 2019, corresponding to 242.414 births.^2^ As all surgical procedures, CS can be complicated by surgical site infections, e.g. wound complications affecting 2% to 7% of women and endometritis in 2% to 16% undergoing CS.^3^ Therefore, a surgical antimicrobial prophylaxis is standard for CS and reduces maternal infectious complications by 60–70%.^4^ Until 2013, prophylactic antibiotics were administered to mothers after the umbilical cord was clamped to prevent antibiotic exposure of the newborn.^5,6^ As large randomized controlled trials and meta-analyses showed a decreased risk for infections when antibiotics are given before the procedure starts,^7–10^ international guidelines changed and now recommend the administration of the antimicrobial prophylaxis 30 to 120 minutes before skin incision.^11,12^ This, however, leads to an intrauterine exposure to antibiotics of all infants born by CS shortly before birth.^13^ Animal models imply that the perinatal exposure to antibiotics has substantial impact on early microbiome establishment of the offspring with potential long-term consequences.^14,15^ For example, prenatal exposure to antibiotics has been associated with an increased risk for childhood asthma, allergies and obesity.^16–18^ Furthermore, antibiotic treatment within the first months of life can influence childhood health including higher incidences for overweight and atopic diseases.^19,20^ There is an urgent need to disentangle the impact of early (and sustained) gut dysbiosis as a potential underlying mechanism for these non-communicable diseases in order to investigate targeted prevention.^21^ While evidence for the influence of perinatal antibiotics on neonatal gut microbiota exists for other settings like intrapartum group B streptococci prophylaxis or prevention of neonatal sepsis,^22–27^ no clear picture has yet been established on how a single dose of surgical antimicrobial prophylaxis before CS affects the early microbiome engraftment.^28^ CS itself is associated with an altered early neonatal gut microbiome resembling maternal skin microbiota compared to vaginal birth, which introduces vaginal microbiota like lactobacilli to the initial gut microbiome.^29^ Differences in the microbiome between cesarean and vaginally born infants remained detectable for the first years of life in some studies.^30,31^ However, among the few studies which also included infants born by CS, conflicting results have been found.^26–28^ The only published trial analyzing the neonatal gut microbiome stratified by the timing of the antibiotic during CS did not find a difference between the study groups.^28^ Furthermore, antimicrobial resistance is an increasing problem in adult and neonatal medicine.^32,33^ Antibiotic resistance genes have been detected in the infant gut as early as days after birth^25,34,35^ and are attributed to perinatal antibiotic exposure and vertical transmission.^23,36^

In our study, we evaluated the effect of timing of antibiotics before skin incision versus after cord clamping on gut microbiome composition and acquisition of antibiotic resistance genes of term born infants born by elective CS in a single center randomized controlled clinical trial.

## Results

### Study population and clinical characteristics

**Figure 1.**
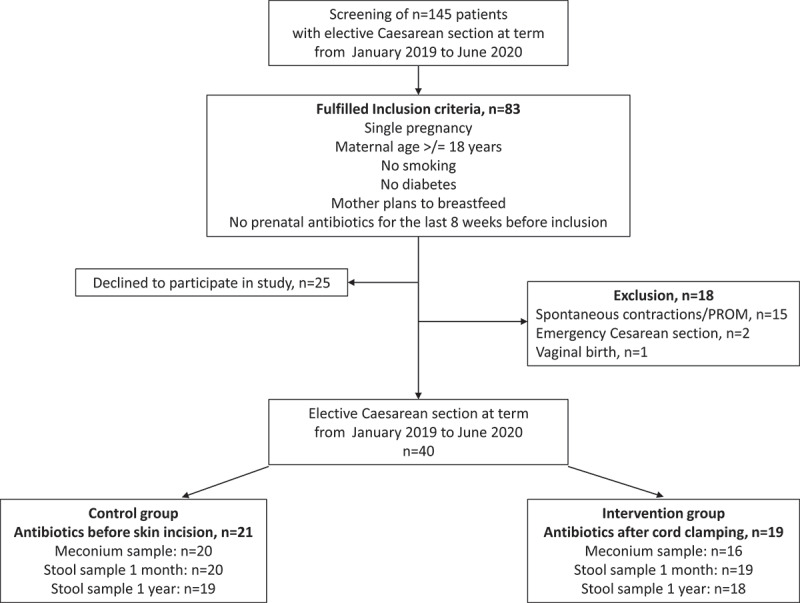
Flowchart of study inclusion.

Between January 2019 and June 2020, 145 pregnant women approaching elective CS were screened in the outpatient clinic. Sixty-two women were excluded for not meeting the inclusion criteria, 25 women declined to participate in the study. Fifty-eight women and their infants were enrolled in the trial (Figure 1). After enrollment, 18 patients had to be excluded because of unforeseen spontaneous labor or premature rupture of membranes (PROM) (n = 15), emergency CS (n = 2) and vaginal birth (n = 1). We included 40 mothers and their infants into the final cohort (n = 21 control group, n = 19 intervention group). One patient from the control group was lost to follow-up 1 month postpartum, n = 39 were included in our data analysis at 1 month. After 1 year, we collected stool samples and clinical data from 37 infants (n = 19 control group, n = 18 intervention group). Both study groups were similar concerning maternal and neonatal characteristics at baseline (Table 1). One month after birth, there was no statistically significant difference between the study groups concerning maternal and neonatal clinical parameters. Two infants had been treated with iv antibiotics in the hospital for a urinary tract infection (ampicillin, cefotaxime, and gentamicin) or a suspected respiratory infection (ampicillin). Postpartum infections were noted in 9/39 mothers, i.e., 3/20 from the control group (1 bronchitis, 2 flu-like symptoms) vs. 6/19 from the intervention group [2 urinary tract infections, 1 tonsillitis, 1 flu-like symptoms and 2 surgical site infections (1 wound infection, 1 endometritis)]. Even though we did not include women into our trial who did not plan to breastfeed prenatally, 2 mothers from the control group and 5 mothers from the intervention group did not breastfeed their infants at month anymore. At 1 year, there were no significant differences of clinical characteristics between the study groups, especially concerning relevant pediatric outcomes like allergies, antibiotic use, and respiratory disease. The total completed months of breastfeeding during the first year were 5.2 and 5.3 months in the two groups (p = .39).

**Table 1.** Clinical characteristics at birth, 1 month and 1 year

	Control group	Intervention group	*p*-value
**AT BIRTH**			
Maternal age at birth	32.1 (4.5)	33.1 (5.9)	0.41
Gravidity	2.9 (1.7)	2.7 (1.3)	0.87
Parity 1+, n (%)	17 (81.0)	15 (78.9)	1.00#
Maternal BMI before pregnancy	24.8 (5.1)	28.7 (9.0)	0.20
Maternal BMI at birth	30.6 (5.0)	33.1 (9.0)	0.70
Gestational age at birth, weeks	38.7 (0.6)	38.7 (0.6)	0.94
Birth weight, grams	3411.2 (354.1)	3463.7 (621.8)	0.66
length at birth, cm	51.2 (2.0)	51.9 (2.0)	0.39
head circumference, cm	35.1 (1.3)	35.4 (1.4)	0.40
Female gender child, n (%)	12 (57.1)	14 (73.7)	0.27*
Umbilical artery pH	7.28 (0.08)	7.30 (0.10)	0.14
Umbilical artery base excess	−3.2 (3.2)	−3.3 (3.2)	0.89
APGAR 1	8.8 (0.8)	8.9 (0.3)	1.00
APGAR 5	9.7 (0.8)	9.7 (0.5)	0.46
APGAR 10	9.8 (0.9)	10.0 (0.2)	0.59
**AT 1 MONTH**			
Breastfeeding only	14 (70.0)	11 (57.9)	0.43*
Bottle only	2 (10.0)	5 (26.3)	0.23#
Breastfeeding + bottle	4 (20.0)	3 (15.8)	1.00#
Antibiotics child since birth	1 (5.0)	1 (5.3)	1.00#
Infection mother	3 (15.0)	6 (31.6)	0.27#
Antibiotics mother	2 (10.0)	5 (26.3)	0.24#
Healing problems scar	1 (5.0)	2 (10.5)	0.61#
Healing problems scar, other than infection	1 (5.0)	1 (5.3)	1.00#
SSI	0 (0.0)	2 (10.5)	0.23#
**AT 1 YEAR**			
Breastfeeding at 1 year, n (%)	5 (26.3)	5 (29.4)	1.00#
breastfeeding, completed months	5.2 (2.9)	5.3 (4.9)	0.39
antibiotic treatment since birth, n (%)	2 (10.5)	3 (17.6)	0.65#
bronchitis since birth, n (%)	2 (10.5)	1 (5.9)	1.00#
allergy, n (%)	0 (0)	3 (17.6)	0.10#
atopic dermatitis, n (%)	2 (10.5)	3 (17.6)	0.65#

Data are given as mean (SD) or n (%). Percentages are given as column percentages. For categorical variables Pearson’s-Chi-square test (*) or Fisher’s exact test (#) and for continuous variables Mann-Whitney-U test were used for calculating statistical significance.

### Timing of antimicrobial prophylaxis impacts the early neonatal gut microbiome and its function

**Figure 2.**
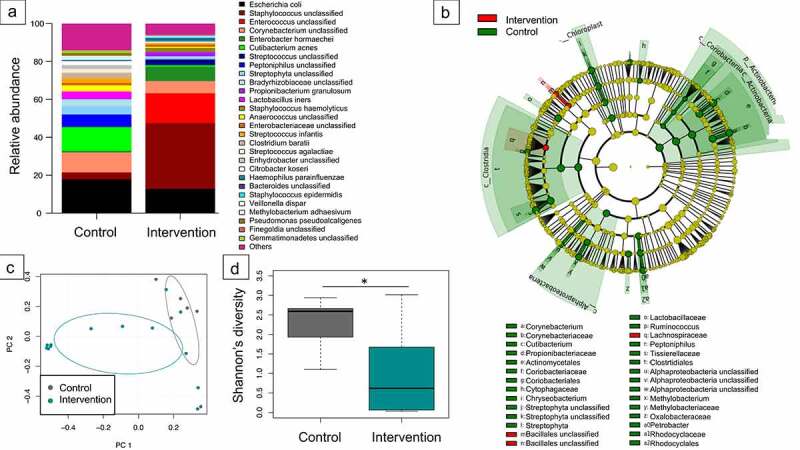
***Microbiome differences in meconium samples stratified to timing of antibiotic prophylaxis.*** Relative abundance of the most abundant genera (a) as well as detected indicator species via linear discriminant analysis effect size (*P*< .05) (b), principal coordinates analysis of beta diversity (permutational multivariate analysis of variance using distance matrices *P* = .026) (c) and Shannon’s diversity index (pairwise Wilcoxon rank sum test * *P* = .035) (d) indicated significant impact of intrapartum antibiotic prophylaxis on neonatal microbiome.

**Figure 3.**
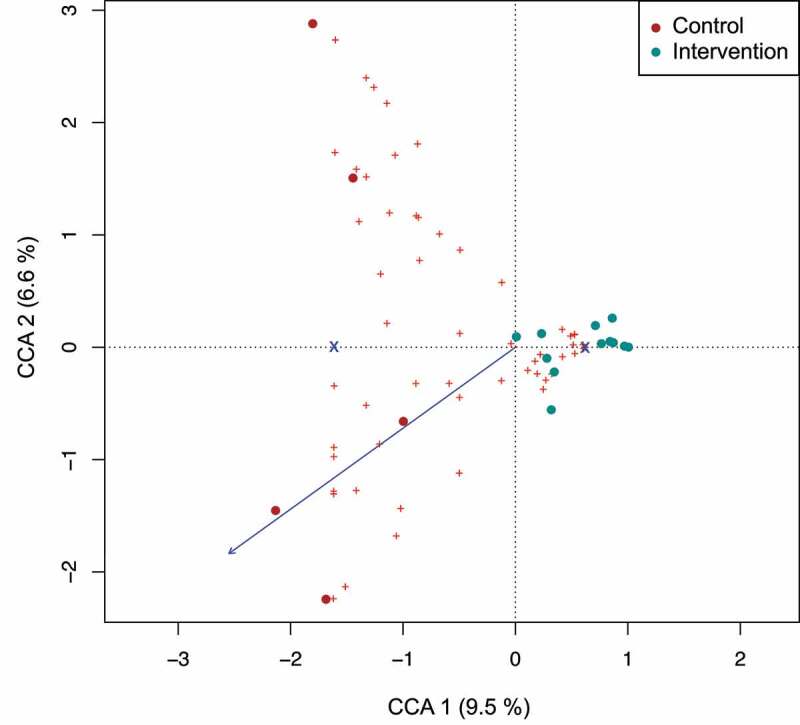
***Interdependence of concentration of antibiotics in cord blood with diversity of infant’s gut microbiome in meconium samples.*** Constrained correspondence analysis with administration of intrapartum antimicrobial prophylaxis set as constrain 1 and concentration of antibiotic in blood as constrain 2 revealed that 6.6% of variation was explained by the concentration of antibiotic in the cord blood suggesting the dependence of changes in microbiome of meconium on the identified concentration of antibiotic.

To follow the development of the infants’ gut microbiome at different time points, 16S rRNA gene sequencing of the V3/V4 hypervariable region was performed. Samples from each time point had a distinct age-specific microbiota composition (Suppl. Figure 1, temporal development). When comparing the relative abundance of taxa in meconium samples, a clear dominance of genus *Staphylococcus* (*p* = .02) was observed for the intervention group (Figure 2a). Control group infants had more diverse microbiota (*p* = .035) (Figure 2d) with higher abundance of the genera *Cutibacterium, Corynebacterium* and *Streptophyta*. Analysis of beta diversity indicated a high degree of dissimilarity between the study groups (*p* = .026) suggesting that timing of antibiotic prophylaxis is critical for the microbial community of infants during their first days of life (Figure 2c). Constrained correspondence analysis revealed that administration of antimicrobial prophylaxis before skin incision accounted for 9.5% of variation (Figure 3). The interdependence between the concentration of cefuroxime in cord blood and microbiota diversity in meconium samples was demonstrated via constrained correspondence analysis which explained 6.6% of the data variability (Figure 3). Furthermore, indicator species analysis revealed numerous species assigned as indicators for the control group: genera *Corynebacterium, Cutibacterium, Chryseobacterium, Ruminococcus, Peptoniphilus, Methylobacterium* and *Petrobacter*, as well as families *Cytophagaceae, Lactobacilaceae, Oxalobacteraceae* and order *Streptophyta* (Figure 2b). Family *Lachnospiraceae* and order *Bacillales* were assigned as indicators for the intervention group.

**Figure 4.**
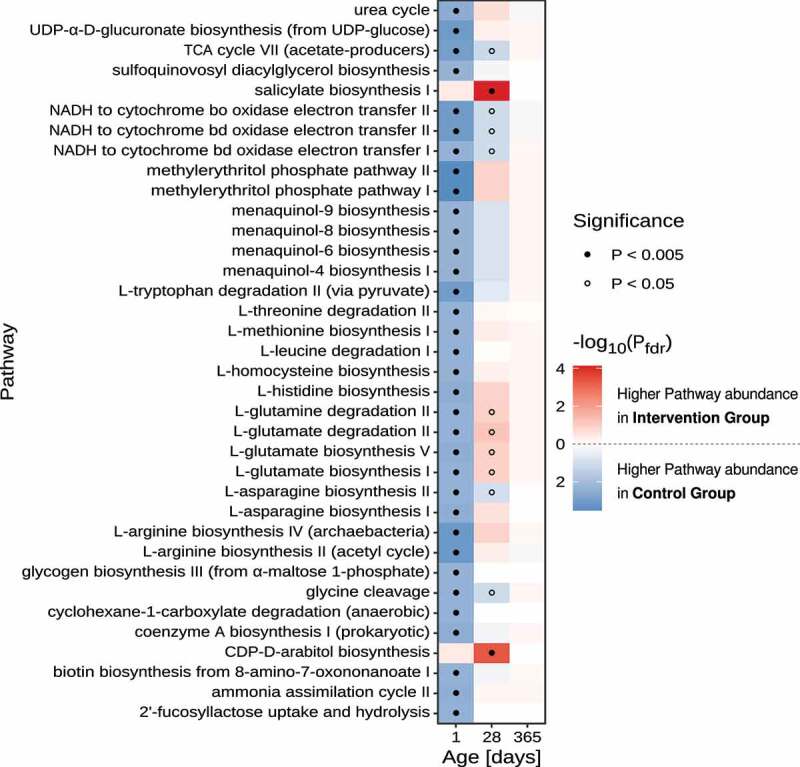
***Differential pathway abundance analyses between control- and intervention group stool samples.*** Heatmap of FDR-adjusted *P*-values refer to the significance levels obtained by comparing the sum of OTU-counts between samples from the control- and intervention group for OTUs that were predicted to harbor the focal metabolic pathway. Only pathways with an FDR-adjusted *P*-value < 0.005 in at least one of the age groups are displayed. Dots indicate pathways with *P*-values below 0.005 (filled circle) and 0.05 (open circles), respectively. Statistical comparison of pathway abundance is based on a zero-inflated beta-binomial (ZIBB) model to account for excessive zeroes and over-dispersion in the sequence count data.^37^

Prediction of the metabolic pathway composition within the microbial communities revealed differential biochemical capacities of the microbiomes from meconium samples between the control and intervention groups (Figure 4). The analysis suggested that several pathways involved in amino acid and nitrogen metabolism are enriched in gut microbial communities from the control group. Those differences are attributed to a combination of diverse taxonomic groups with the largest contribution by members of the genera *Escherichia, Cutibacterium* (formerly part of the genus *Propionibacterium*), and *Corynebacterium* (Suppl. Figure 3). Interestingly, also genes involved in the pathway for 2’-fucosyllactose (short: 2-FL; a human milk oligosaccharide) uptake and utilization were enriched in the control group, which is attributed to OTUs that were mapped to a genome from the species *Cutibacterium acnes* (Figure 4 and Suppl. Figure 2). The pathway consists of two steps: First, the uptake of 2-FL through an ATP-binding cassette (ABC) transporter, and second, the hydrolysis of 2-FL by a 1,2-alpha-L-fucosidase (EC: 3.2.1.63). To our best knowledge, the ability to degrade 2-FL has not yet been associated with *C. acnes*. In order to scrutinize that the prediction of 2-FL uptake and hydrolysis is not a false-positive prediction due to a potential contamination in the specific genome sequence of the representative species-level genome bin for *C. acnes*, we re-analyzed all 22 *C. acnes* genomes from the UHGG collection using gapseq to predict the presence/absence of this pathway. For 20 of those genomes, gapseq found genes that are similar (bitscore > 137, E-value < 10^−37^) to reference sequences for 1,2-alpha-L-fucosidase (EC: 3.2.1.63) from the Uniprot database. Putative genes for the ABC-transporter for 2-FL were found in 21 out of the 22 *C. acnes* genomes. Taken together, these results suggest that putative genes for the utilization of 2-FL are conserved across strains of *C. acnes*. At the age of 1 month, fewer significant differences in metabolic pathway composition were observed between the study groups (Figure 4 and Suppl. Figure 3).

### Timing of antimicrobial prophylaxis for cesarean section and effects on microbiome establishment during infancy

**Figure 5.**
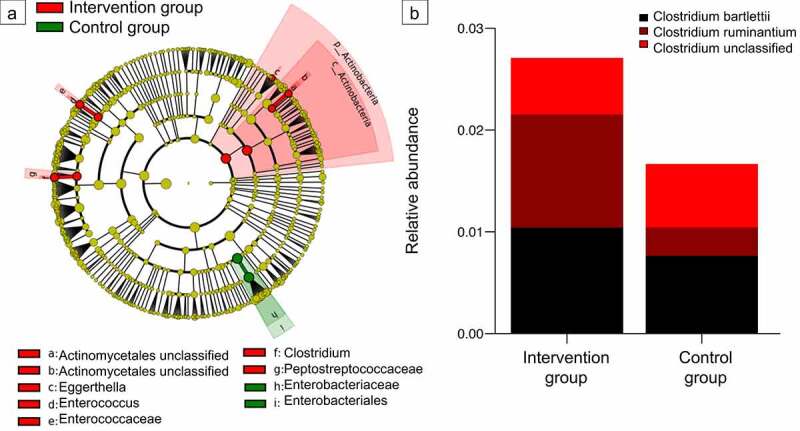
***Impact of intrapartum antimicrobial prophylaxis on the gut microbiome at later stages of microbiome development.*** (a) Indicator species identified via linear discriminant analysis effect size (*P* < .05) for intervention and control group infants at the age of 1 month. (b) Relative abundance of the genus *Clostridium* from the family *Peptostreptococcaceae* (found to be associated with the intervention group at 1 month) is still increased at the time point of 1 year without reaching statistical significance (Wilcoxon rank-sum test).

Comparison of microbiome diversity and composition at the age of 1 month and 1 year revealed that the strong effect of timing of antimicrobial prophylaxis on the gut microbiota diminished over time. Global differences in alpha and beta diversity at these time points were not significant (Suppl. Figure 4). However, indicator species analysis disclosed that some changes remained detectable after 1 month. Of note, some *Clostridium* species belonging to the *Peptostreptococcaceae* family (associated to intervention group at on month) tended to be still more abundant in the intervention group throughout the first year of life, which was not significant (Wilcoxon rank-sum test, *p* > .05 for all taxa) (Figure 5a, b).

### Acquisition of antibiotic resistance genes starts in the first days of life

**Figure 6.**
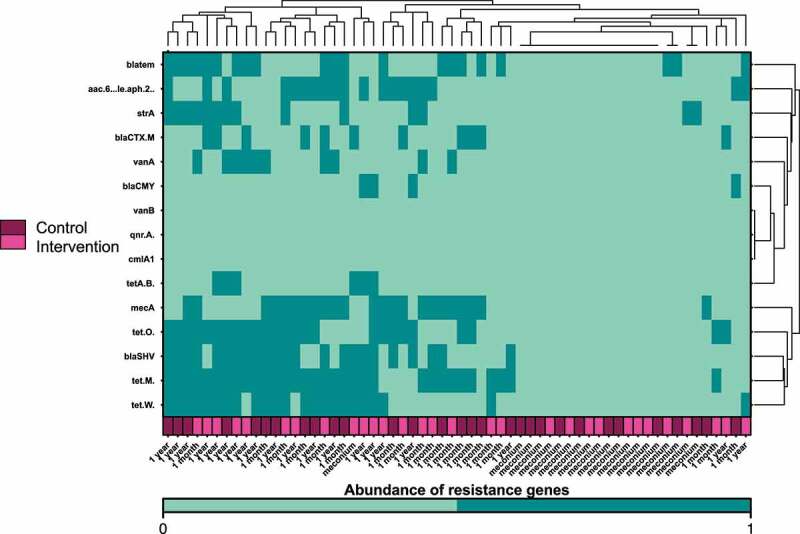
***Heatmap and hierarchical clustering according to the abundance of antibiotic resistance genes on the first days of life, at one month and at one year after birth.*** Timing of antibiotic prophylaxis was selected as covariate for the analysis. Clustering was performed based on the Euclidean distance; dark green represents abundance of the resistance genes and aquamarine the absence.

To evaluate the abundance of selected antibiotic resistance genes in the gut microbiome of infants, a subset of 10 samples from each time point was analyzed via PCR with specific primers. While in meconium samples detection of selected resistance genes was sparse (with one exception), 10 and 11 of 15 tested resistance genes were identified at the age of 1 month and 1 year, respectively (Figure 6). Six of the analyzed antibiotic resistance genes were found in more than 35% of the samples, among them three genes encoding resistance against tetracyclines (*tet(W), tet(M), tet(O)*), two beta lactamase genes (*blaSHV, bla tem*) and *mecA* encoding transpeptidase facilitating resistance against penicillin-like antibiotics. Of note, the microbiome of one child treated with ampicillin, cefotaxim und gentamicin within its first month of life had developed resistance genes for penicillins and aminoglycoside antibiotics. While we observed variation of resistance gene prevalence within and between the groups, we were not able to demonstrate a link to the timing of antimicrobial prophylaxis.

## Discussion

We performed a randomized controlled trial on the timing of antimicrobial prophylaxis in 40 women undergoing elective primary CS and its effects on the neonatal microbiome development. Control infants were exposed to a single dose of cefuroxime, as confirmed by relevant cord blood concentrations, while in the intervention group cefuroxime was administered after cord clamping. Timing of antimicrobial prophylaxis significantly influenced the early microbiome, specifically we observed: (i) predominance of *Staphylococcus* (indicator family *Lachnospiraceae*) in meconium samples of the intervention group, (ii) predominance of the genera *Cutibacterium, Corynebacterium* and *Streptophyta*, higher diversity and increased 2’-fucosyllactose metabolism in meconium samples of the control group, (iii) shifts in diversity related to cefuroxime concentrations and (iv) acquisition of antibiotic resistance genes starting in the first days of life regardless of antibiotic exposure.

Recent data suggest that both, mode of delivery and perinatal antibiotic exposure, have an impact on infants’ gut microbiome establishment and long-term health, e.g. risk for asthma, inflammatory diseases and obesity.^38^ Microbiota development patterns throughout infancy in our study resembled the results from others^39^ and are typical for infants born via CS.^29,40^ In comparison to samples collected in the first days of life, 1 month samples exhibited increased abundance of *Bifidobacteria* known to be associated with human milk microbiota.^41,42^ Elevated abundance of genus Bacteroides observed at the age of 1 year goes in line with studies indicating that upon the introduction of solid food the composition of the infants’ microbiome becomes more similar with the adult one.^43^

In our cohort, the greatest differences in microbial composition and significant changes in diversity between the study groups were present during the first days of life. Here, the impact on beta-diversity was correlated with the cefuroxime levels which are more slowly eliminated in neonates than in adults.^13^ Numerous indicator species, e.g., *Cytophagaceae, Lactobacilaceae, Oxalobacteraceae*, were assigned to the control group. These skin microbiome-related species^29,44^ and taxa have been previously linked to infectious and inflammatory diseases^45^ which may translate into an early acquisition of a microbial risk signature. On the contrary, the indicator family *Lachnospiraceae* in the intervention group is associated with protection against *Clostridium difficile* infection^46^ and decreased risk of asthma and intestinal permeability in neonates.^43,47^ Furthermore, the functional potential of the microbiome may be critically influenced by timing antimicrobial prophylaxis. Of special interest appears the putative presence of genes in *C. acnes* encoding for the utilization of 2-fucosyllactose, a major human milk oligosaccharide (HMO) in combination with the observation that the abundance of *C. acnes* is higher in meconium samples from the control group compared to the intervention group. The presence of HMOs in human milk has important anti-infective properties and stimulates the immune system. HMOs are the main energy source of early infant gut colonizing *Bifidobacteria* (e.g., *Bifidobacterium longum* subsp. *infantis*), which helps to outcompete pathogenic bacteria. *Bifidobacteria* produce immune-stabilizing short chain fatty acids from HMOs and are therefore a candidate for probiotic supplementation.^48^ Hence, increased early degradation of such HMOs in the control group by bacteria other than from the genus *Bifidobacterium* may decrease the functional capacity of the microbiome and hamper the engraftment of beneficial *Bifidobacteria*. In addition, the differential pathway abundance analysis also showed a higher frequency of pathways involved in amino acid metabolism in the control group. This observation further suggests that the timing of antimicrobial prophylaxis has a strong impact on the capacity of the gut microbiome to utilize nutrients from the milk diet.

In the intervention group, we noted a sustained abundance of the genus *Clostridium* during infancy which is associated with immune-protective and anti-inflammatory effects, e.g. allergy and sepsis protection.^49,50^ The absence of *Clostridium* in the control infants’ microbiome of our population may indicate an additional hallmark of the microbial risk signature.^51,52^

There is evidence of a long-lasting impact of intrapartum exposure to antibiotics on the diversity and composition of the gut microbiome from studies on vaginally born infants with group B *Streptococci* positive mothers.^22–24^ However, among the few studies on infants born by CS, conflicting results have been published, including lack of identified microbiota changes,^28^ results contrary to ours,^26^ and results partially in line with ours.^27^ In our study, strict selection criteria were applied for women undergoing CS (e.g., no labor, no preexisting disease or antibiotic treatment, planned breastfeeding). In addition, differences to previous studies may be related to sampling strategy, methodological adjustments including metabolic prediction models and consideration of the antibiotic used during CS.^53^ Taking all this into account, our study supports the hypothesis that the current standard of timing antimicrobial prophylaxis before skin incision leads to significant effects on the early developing microbiome with a yet undefined impact on long-term outcome.

There are several study limitations. We conducted a small-scale pilot study with follow-up of 1 year after CS. Despite the effort to enroll a rather homogenous population, we might have missed potential confounding variables. The differences we found in the neonatal gut microbiome were predominantly present in meconium samples which are lower in microbial content than fecal samples beyond the first days of life. Furthermore, it is possible that modest differences seen in meconium samples are subsequently resolved and potentially inconsequential with respect to clinical outcomes, such as allergies, asthma, or obesity. To adjust for additional genetic or environmental risk factors, a larger trial with a longer follow-up would be necessary. Moreover, we did not focus on the impact of breast feeding versus formula feeding on the infant microbiome. In our study design, we excluded pregnant women who did not plan to breastfeed. By enrollment we achieved an acceptable homogeneity with regard to breastfeeding, as only seven mothers did not breastfeed anymore at 1 month and seven mothers partially used formula. The total completed months of breastfeeding during the first year were 5.2 and 5.3 months in the two groups (*p* = .39, see Table 1). Furthermore, the impact of breastfeeding on gut microbiomes was not significant in constrained correspondence analysis (explained proportion: 0.0205, *p*-value: 0.77).

On the maternal side, there were 2 SSIs in the intervention group versus none in the control group, which did not reach statistical significance. Although the SSIs were not critical and could be treated with antibiotics, they might have been prevented by a surgical antimicrobial prophylaxis before skin incision. This would be in line with previous studies showing a higher risk for endometritis and wound infections when antibiotics are administered after cord clamping,^7–10^ which lead to the current international recommendations to apply perioperative antibiotics 30 to 120 minutes before skin incision.^11,12^ However, a recently published large prospective multicenter analytic study from Switzerland including 55.901 women did not find an increased risk for SSI in mothers who received the surgical antimicrobial prophylaxis after cord clamping. It was the largest clinical study on the topic so far.^54^ The prevention of maternal infectious complications is highly important. SSIs after birth pose a health risk to mothers, interfere with maternal care for the newborn and often make post-partum antibiotics necessary which can transfer to the infant via breast milk. Based on the conflicting results of trials on the impact of timing of the surgical antimicrobial prophylaxis on maternal SSIs, more data is needed with respect to specific obstetric settings (elective versus emergency CS, facility hygiene standards, postpartum standard care).

Our data also support the need for antibiotic stewardship programs in perinatal medicine, as variable antibiotic resistance genes are acquired already in the first days of life.^34^ We confirmed the presence of tetracycline, beta lactam and aminoglycoside resistance genes in the infant gut microbiome.^25,55^ Given the exploratory design of our study, we did not detect differences in the antibiotic resistance patterns based on exposure to antimicrobial prophylaxis, as it has been previously described.^25^ Considering that the gut microbiome serves as a reservoir of antibiotic resistance genes, any reduction of early antibiotic exposure is likely to improve the susceptibility to antimicrobial therapy.

In conclusion, we performed small-scale a randomized controlled trial with well-defined selection criteria of women undergoing elective CS, follow-up of infants during infancy and the use of metabolic prediction models from 16S sequencing data of microbiome samples. Our study is exploratory due to limited size and single-center design. We propose that timing of antimicrobial prophylaxis is critical for early microbiome engraftment in term born infants. We propose that cutting-edge multi-omics techniques and systems biology approaches can help to revisit previous risk-benefit analyses of timing antimicrobial prophylaxis. The design of future large clinical trials with adequate consideration of infant outcomes suggests potential targets of microbiome modification to the benefit of the host, e.g., next-generation pro- and synbiotics.

## Methods

### Study population and ethics

This exploratory randomized controlled trial was performed at the University Hospital of Lübeck, Germany, from 1/2019 to 6/2020. Approval by the local ethics committee for research in human subjects of the University of Lübeck has been granted on October 9^th^, 2018 (Reference number 18–264). All parents provided written informed consent prior to CS. The study was registered at the German Registry for clinical studies (DRKS), no. DRKS00025305.

#### Population:

We included 40 pregnant women undergoing elective CS after 37 completed weeks of gestation. Inclusion criteria were age ≥ 18 years, planned breastfeeding and written informed consent by both pregnant woman and partner. Exclusion criteria were signs of spontaneous labor or rupture of membranes at the time of CS, emergency CS, multiple pregnancy, allergy against cephalosporines, smoking, gestational diabetes and preexisting diabetes, and the use of systemic antibiotics within 8 weeks prior CS.

#### Control/Intervention:

Study participants were randomly assigned to receive cefuroxime 1500 mg intravenously 30 minutes before skin incision (control group, n = 21) or immediately after cord clamping (intervention group, n = 19). To assign the mother-infant pairs to a study arm, a randomization sequence was created before the inclusion of the first patient. On the day of the CS, the mother was treated as with the antibiotic before skin incision or after cord-clamping, based on the randomization sequence.

#### Outcomes:

The primary endpoint was microbiome composition and prediction of corresponding metabolic function in meconium samples of infants as determined by 16S rRNA gene sequencing. Secondary endpoints were microbiome composition at 1 month and 1 year of life, antibiotic resistome patterns, risk for surgical site infection of the mother (e.g., endometritis and wound infection) and infections of the infant.

### Sample collection

Prior to skin disinfection, a rectal swab was taken from the pregnant woman in the operation theater. Cord blood was collected during CS after cord clamping from infants who received the antibiotic before skin incision to measure the antibiotic concentration by high-performance liquid chromatography. A meconium sample was collected from all infants during the first days of life as soon as meconium was passed. A fecal sample was collected from all infants at the age of 1 month (sampling 28–31 postnatal days) and 1 year (sampling 10–12 months). The samples were stored in the refrigerator at −20°C immediately after collection and transported in cool boxes to −80°C within 72 hours. To assure quality of sample preservation, the follow-up fecal samples at 1 month and 1 year of age were picked up from home environment by study personnel. A standardized questionnaire about diet, wound healing, surgical site infections and daily habits at both timepoints as well as infants’ outcomes, in particular infections (local or systemic, need for antibiotic treatments) was provided by the families at 1 month and 1 year.

### Microbial DNA isolation

Fecal samples were thawed and approximately 100 mg of stool samples or 200 mg of meconium samples were processed using DNeasy® PowerSoil® DNA Isolation Kit (Qiagen GmbH, Hilden, Germany). 500 µl of phosphate buffered saline were added to rectal swabs and vortexed for 5 minutes prior to processing. A negative extraction control was carried out with each round of isolation. Thereafter, DNA was stored at −20°C until further usage.

### Polymerase chain reaction amplification and 16S rRNA gene sequencing

Partial sequences of the 16S rRNA gene of isolated DNA samples were amplified using linker and indices-containing primers targeting V3/V4 hypervariable regions of the 16S rRNA gene which we have optimized^56^ based on Fadrosh et al.^57^ Primer sequences are given with the supplement (Supplementary table 1). Polymerase chain reaction (PCR) was carried out in accordance with the following parameters: 98°C for 30 seconds, 30 cycles with 98°C for 9 seconds, 55°C for 60 seconds and 72°C for 90 seconds, final step was set to 72°C for 10 minutes. Amplicons were stored at −20°C until further usage. Concentration of the amplicons was estimated via agarose gel electrophoresis using GeneRuler 100 bp DNA Ladder (Thermo Fischer Scientific, Waltham, USA) as a reference. Equimolar amounts of each amplicon were pooled, ran on an agarose gel, and purified using MinElute® Gel Extraction Kit (Qiagen GmbH, Hilden, Germany). Sequencing was performed using MiSeq® platform (Illumina®, San Diego, California, USA) and MiSeq® reagent Kit V3 for 600 cycles using PhiX library as a positive control. Negative extraction controls were incorporated to ensure lack of reagents contamination. Only samples giving a clearly definable amplicon after PCR were subjected to data processing and statistical analysis while isolation controls remained negative.

### Bioinformatics and statistical analysis

Fastq files were processed using mothur version 1.44.1.^58,59^ Sequences with homopolymers of more than 12 bases or sizes longer than 500 bp were removed. Remaining sequences were aligned against mothur’s SILVA reference data base,^60^ not aligned sequences were excluded from further analysis. Chimeric sequences were identified and removed using VSEARCH algorithm.^61^ Taxonomic assignment was conducted using Greengenes Data Base,^62^ mitochondrial, archaeal and eukaryotic sequences were removed. Operational taxonomic units-based analysis was performed on a random subset of 1800 reads/sample with cutoff level of 0.03 or based on taxonomic assignment. Statistical analysis and graphical visualization were obtained using R (version 4.0.1). Alpha diversity of the microbiome was analyzed via Shannon’s diversity and Shannon’s evenness indexes, as well as by calculating the number of species and Chao 1 index by vegan package in R.^63^ Differences between groups were calculated using non-parametric pairwise Wilcoxon rank-sum test. Beta diversity analysis was performed using principal coordinates analysis based on Bray-Curtis dissimilarity metric (vegan^63^ and labdsv package in R^64^). To assess the contribution of specific parameters (e.g. antibiotic concentration) a constrained correspondence analysis (vegan package in R^63^) was performed, which specifically addresses the variances of the data set explained by the respective variables. Analysis of variance like permutation test and permutational multivariate analysis of variance using distance matrices were used to estimate the difference between groups. Identification of indicator species was performed by multi-level pattern analysis via the indicspecies package in R^65^ and Linear Discriminant Analysis Effect Size provided by Galaxy Project Platform.^66,67^ We customized analysis input by adapting the correct genus assignment for *Cutibacterium acnes*.

For the clinical parameters, descriptive statistics using percentages for peri- and postnatal parameters were carried out. For categorical variables Pearson’s Chi-square test or Fisher’s exact test were used while and for continuous variables Mann-Whitney-U test was applied to calculate statistical significance. The type I error level was set to 0.05. All statistical analyses on clinical parameters were performed with SPSS 26.0 software (IBM SPSS Statistics for Windows, Version 26.0. Munich, Germany).

### Prediction of metabolic pathway composition

Representative 16S sequences of the calculated OTUs from this study were mapped to the 16S rRNA gene sequences included in the 4,644 species-level prokaryotic genome bins from the Unified Human Gastrointestinal Genome (UHGG) collection.^68^ The mapping was based on pairwise alignments using USEARCH^69^ and the highest sequence identity score with a minimum identity of 97% and a minimum query sequence coverage of 95%. The presence of metabolic pathways in organisms from the UHGG collection that were part of the mapping was predicted using gapseq v1.1.^70^ In detail, the pathway prediction was facilitated by the ‘gapseq find’ module using the options ‘-p all’ and ‘-m Bacteria’ to search for all pathways from the MetaCyc database^71^ and gapseq’s additional pathways that were described for bacteria. Based on the mapping of OTUs to UHGG and the pathway predictions, the OTU-count table was translated into a Pathway count table by summing up the count of OTUs that were mapped to UHGG Species, which were predicted to have the focal individual pathways.

### Analysis of differential pathway abundance

Like the OTU count table, the pathway count table also represents sparse discrete data. To identify differences in the pathway count distribution between sample groups (control- and intervention group), a zero-inflated beta-binomial (ZIBB) model was employed by using the R-package ‘ZIBBSeqDiscovery’.^37^ We tested differential pathway abundance between the control- and intervention groups while correcting for the potential effect of differences in the total pathway count per sample.

### Detection of antibiotic resistance genes in fecal samples

A randomly selected subset of matching 20 meconium samples, 20 stool samples collected from infants at 1 month and 20 stool samples collected from infants at 1 year of age was screened for the presence of 15 selected antibiotic resistance genes. Detection of antibiotic resistance genes was performed using PCR with specific primers (Supplementary table 2).^25,72–74^ The following parameters were applied: 94°C for 5 minutes, 35 cycles of 94°C for 30 seconds, 30 seconds set to primer-specific annealing temperature, 60 seconds at 72°C, end cycle for 7 minutes at 72°C. Agarose gel electrophoresis using GeneRuler 100 bp Plus DNA Ladder as a marker was performed to identify the presence of resistance genes in the samples. Pearson’s Chi-square test incorporated in stats package in R^75^ was used to analyze inter-group differences. Heatmap was created using BoutrosLab.plotting.general package in R.^76^ Clustering was performed based on the Euclidean distance estimation.

## Supplementary Material

Supplemental Material

## Figures and Tables

**Table:** 

ABC	ATP-binding cassette
CS	cesarean section
DNA	desoxyribonucleic acid
DRKS	German Registry for clinical studies
2-FL	2’-Fucosyllactose
HMO	human milk oligosaccharide
OR	odds ratio
Otu	operational taxonomic unit
PCR	polymerase chain reaction
PROM	premature rupture of membranes
rRNA	ribosomal ribonucleic acid
SSI	surgical site infection
UHGG	Unified Human Gastrointestinal Genomes
ZIBB	zero-inflated beta-binomial

## Data Availability

The data that support the findings of this study are openly available in European Nucleotide Archive at https://www.ebi.ac.uk/ena/browser/home, reference number PRJEB47587.
