# Supplementary material for: Timing of antimicrobial prophylaxis for cesarean section is critical for gut microbiome development in term born infants
Source: Gut Microbes. 2022 Feb 20;14(1):2038855. doi: 10.1080/19490976.2022.2038855 (PMC8865290; doi:10.1080/19490976.2022.2038855)
Supplement: Supplemental Material [file KGMI_A_2038855_SM3517.docx]

**Supporting Information**

**Supplementary table 1: Primers used for amplification of the V3/V4 hetero region of microbial 16S rRNA gene for next generation sequencing**

| Number | Oligo name | Sequence 5’-3’ | Index |
| --- | --- | --- | --- |
| 1 | hV3F_MID1 | AATGATACGGCGACCACCGAGATCTACAC ATCGTACG ACACTCTTTCCCTACACGACGCTCTTCCGATCT CCTACGGGAGGCAGCAG | ATCGTACG |
| 2 | hV3F_MID2 | AATGATACGGCGACCACCGAGATCTACAC ACTATCTG ACACTCTTTCCCTACACGACGCTCTTCCGATCT T CCTACGGGAGGCAGCAG | ACTATCTG |
| 3 | hV3F_MID3 | AATGATACGGCGACCACCGAGATCTACAC TAGCGAGT ACACTCTTTCCCTACACGACGCTCTTCCGATCT GT CCTACGGGAGGCAGCAG | TAGCGAGT |
| 4 | hV3F_MID4 | AATGATACGGCGACCACCGAGATCTACAC CTGCGTGT ACACTCTTTCCCTACACGACGCTCTTCCGATCT CGA CCTACGGGAGGCAGCAG | CTGCGTGT |
| 5 | hV3F_MID5 | AATGATACGGCGACCACCGAGATCTACAC TCATCGAG ACACTCTTTCCCTACACGACGCTCTTCCGATCT ATGA CCTACGGGAGGCAGCAG | TCATCGAG |
| 6 | hV3F_MID6 | AATGATACGGCGACCACCGAGATCTACAC CGTGAGTG ACACTCTTTCCCTACACGACGCTCTTCCGATCT TGCGA CCTACGGGAGGCAGCAG | CGTGAGTG |
| 7 | hV3F_MID7 | AATGATACGGCGACCACCGAGATCTACAC GGATATCT ACACTCTTTCCCTACACGACGCTCTTCCGATCT GAGTGG CCTACGGGAGGCAGCAG | GGATATCT |
| 8 | hV3F_MID8 | AATGATACGGCGACCACCGAGATCTACAC GACACCGT ACACTCTTTCCCTACACGACGCTCTTCCGATCT CCTACGGGAGGCAGCAG | GACACCGT |
| 9 | hV3F_MID9 | AATGATACGGCGACCACCGAGATCTACAC CTACTATA ACACTCTTTCCCTACACGACGCTCTTCCGATCT T CCTACGGGAGGCAGCAG | CTACTATA |
| 10 | hV3F_MID10 | AATGATACGGCGACCACCGAGATCTACAC CGTTACTA ACACTCTTTCCCTACACGACGCTCTTCCGATCT GT CCTACGGGAGGCAGCAG | CGTTACTA |
| 11 | hV3F_MID11 | AATGATACGGCGACCACCGAGATCTACAC AGAGTCAC ACACTCTTTCCCTACACGACGCTCTTCCGATCT CGA CCTACGGGAGGCAGCAG | AGAGTCAC |
| 12 | hV3F_MID12 | AATGATACGGCGACCACCGAGATCTACAC TACGAGAC ACACTCTTTCCCTACACGACGCTCTTCCGATCT ATGA CCTACGGGAGGCAGCAG | TACGAGAC |
| 13 | hV3F_MID13 | AATGATACGGCGACCACCGAGATCTACAC ACGTCTCG ACACTCTTTCCCTACACGACGCTCTTCCGATCT TGCGA CCTACGGGAGGCAGCAG | ACGTCTCG |
| 14 | hV3F_MID14 | AATGATACGGCGACCACCGAGATCTACAC TCGACGAG ACACTCTTTCCCTACACGACGCTCTTCCGATCT GAGTGG CCTACGGGAGGCAGCAG | TCGACGAG |
| 15 | hV3F_MID15 | AATGATACGGCGACCACCGAGATCTACAC GATCGTGT ACACTCTTTCCCTACACGACGCTCTTCCGATCT CCTACGGGAGGCAGCAG | GATCGTGT |
| 16 | hV3F_MID16 | AATGATACGGCGACCACCGAGATCTACAC GTCAGATA ACACTCTTTCCCTACACGACGCTCTTCCGATCT T CCTACGGGAGGCAGCAG | GTCAGATA |
| 17 | hV3F_MID17 | AATGATACGGCGACCACCGAGATCTACAC ACGACGTG ACACTCTTTCCCTACACGACGCTCTTCCGATCT GT CCTACGGGAGGCAGCAG | ACGACGTG |
| 18 | hV3F_MID18 | AATGATACGGCGACCACCGAGATCTACAC CGTCGCTA ACACTCTTTCCCTACACGACGCTCTTCCGATCT CGA CCTACGGGAGGCAGCAG | CGTCGCTA |
| 19 | hV3F_MID19 | AATGATACGGCGACCACCGAGATCTACAC GCTCTAGT ACACTCTTTCCCTACACGACGCTCTTCCGATCT ATGA CCTACGGGAGGCAGCAG | GCTCTAGT |
| 20 | hV3F_MID20 | AATGATACGGCGACCACCGAGATCTACAC TGCGTACG ACACTCTTTCCCTACACGACGCTCTTCCGATCT TGCGA CCTACGGGAGGCAGCAG | TGCGTACG |
| 1 | hV4R_MID_A | CAAGCAGAAGACGGCATACGAGAT AACTCTCG GTGACTGGAGTTCAGACGTGTGCTCTTCCGATCT GGACTACHVGGGTWTCTAAT | CGAGAGTT |
| 2 | hV4R_MID_B | CAAGCAGAAGACGGCATACGAGAT ACTATGTC GTGACTGGAGTTCAGACGTGTGCTCTTCCGATCT A GGACTACHVGGGTWTCTAAT | GACATAGT |
| 3 | hV4R_MID_C | CAAGCAGAAGACGGCATACGAGAT AGTAGCGT GTGACTGGAGTTCAGACGTGTGCTCTTCCGATCT TC GGACTACHVGGGTWTCTAAT | ACGCTACT |
| 4 | hV4R_MID_D | CAAGCAGAAGACGGCATACGAGAT CAGTGAGT GTGACTGGAGTTCAGACGTGTGCTCTTCCGATCT CTA GGACTACHVGGGTWTCTAAT | ACTCACTG |
| 5 | hV4R_MID_E | CAAGCAGAAGACGGCATACGAGAT CGTACTCA GTGACTGGAGTTCAGACGTGTGCTCTTCCGATCT GATA GGACTACHVGGGTWTCTAAT | TGAGTACG |
| 6 | hV4R_MID_F | CAAGCAGAAGACGGCATACGAGAT CTACGCAG GTGACTGGAGTTCAGACGTGTGCTCTTCCGATCT ACTCA GGACTACHVGGGTWTCTAAT | CTGCGTAG |
| 7 | hV4R_MID_G | CAAGCAGAAGACGGCATACGAGAT GGAGACTA GTGACTGGAGTTCAGACGTGTGCTCTTCCGATCT TTCTCT GGACTACHVGGGTWTCTAAT | TAGTCTCC |
| 8 | hV4R_MID_H | CAAGCAGAAGACGGCATACGAGAT GTCGCTCG GTGACTGGAGTTCAGACGTGTGCTCTTCCGATCT GGACTACHVGGGTWTCTAAT | CGAGCGAC |
| 9 | hV4R_MID_I | CAAGCAGAAGACGGCATACGAGAT GTCGTAGT GTGACTGGAGTTCAGACGTGTGCTCTTCCGATCT A GGACTACHVGGGTWTCTAAT | ACTACGAC |
| 10 | hV4R_MID_J | CAAGCAGAAGACGGCATACGAGAT TAGCAGAC GTGACTGGAGTTCAGACGTGTGCTCTTCCGATCT TC GGACTACHVGGGTWTCTAAT | GTCTGCTA |
| 11 | hV4R_MID_K | CAAGCAGAAGACGGCATACGAGAT TCATAGAC GTGACTGGAGTTCAGACGTGTGCTCTTCCGATCT CTA GGACTACHVGGGTWTCTAAT | GTCTATGA |
| 12 | hV4R_MID_L | CAAGCAGAAGACGGCATACGAGAT TCGCTATA GTGACTGGAGTTCAGACGTGTGCTCTTCCGATCT GATA GGACTACHVGGGTWTCTAAT | TATAGCGA |
| 13 | hV4R_MID_M | CAAGCAGAAGACGGCATACGAGAT AAGTCGAG GTGACTGGAGTTCAGACGTGTGCTCTTCCGATCT ACTCA GGACTACHVGGGTWTCTAAT | CTCGACTT |
| 14 | hV4R_MID_N | CAAGCAGAAGACGGCATACGAGAT ATACTTCG GTGACTGGAGTTCAGACGTGTGCTCTTCCGATCT TTCTCT GGACTACHVGGGTWTCTAAT | CGAAGTAT |
| 15 | hV4R_MID_O | CAAGCAGAAGACGGCATACGAGAT CATAGAGA GTGACTGGAGTTCAGACGTGTGCTCTTCCGATCT GGACTACHVGGGTWTCTAAT | TCTCTATG |
| 16 | hV4R_MID_P | CAAGCAGAAGACGGCATACGAGAT CGTAGATC GTGACTGGAGTTCAGACGTGTGCTCTTCCGATCT A GGACTACHVGGGTWTCTAAT | GATCTACG |
| 17 | hV4R_MID_Q | CAAGCAGAAGACGGCATACGAGAT GCGCACGT GTGACTGGAGTTCAGACGTGTGCTCTTCCGATCT TC GGACTACHVGGGTWTCTAAT | ACGTGCGC |
| 18 | hV4R_MID_R | CAAGCAGAAGACGGCATACGAGAT GGTACTAT GTGACTGGAGTTCAGACGTGTGCTCTTCCGATCT CTA GGACTACHVGGGTWTCTAAT | ATAGTACC |
| 19 | hV4R_MID_S | CAAGCAGAAGACGGCATACGAGAT TACGAGCA GTGACTGGAGTTCAGACGTGTGCTCTTCCGATCT GATA GGACTACHVGGGTWTCTAAT | TGCTCGTA |
| 20 | hV4R_MID_T | CAAGCAGAAGACGGCATACGAGAT TCAGCGTT GTGACTGGAGTTCAGACGTGTGCTCTTCCGATCT ACTCA GGACTACHVGGGTWTCTAAT | AACGCTGA |
| 21 | hV4R_MID_U | CAAGCAGAAGACGGCATACGAGAT AGCTGCTA GTGACTGGAGTTCAGACGTGTGCTCTTCCGATCT TTCTCT GGACTACHVGGGTWTCTAAT | TAGCAGCT |
| 22 | hV4R_MID_V | CAAGCAGAAGACGGCATACGAGAT CTCGTTAC GTGACTGGAGTTCAGACGTGTGCTCTTCCGATCT GGACTACHVGGGTWTCTAAT | GTAACGAG |
| 23 | hV4R_MID_W | CAAGCAGAAGACGGCATACGAGAT GTATACGC GTGACTGGAGTTCAGACGTGTGCTCTTCCGATCT A GGACTACHVGGGTWTCTAAT | GCGTATAC |
| 24 | hV4R_MID_X | CAAGCAGAAGACGGCATACGAGAT TCGCTACG GTGACTGGAGTTCAGACGTGTGCTCTTCCGATCT TC GGACTACHVGGGTWTCTAAT | CGTAGCGA |

**Supplementary table 2: Primers used for identification of antibiotic resistance genes**

| **Gene** | **Oligonucleotide** | | **Annealing T** | **Awaited size, bp** | **Antibiotic group** | **Reference** |
| --- | --- | --- | --- | --- | --- | --- |
|  | **Forward** | **Reverse** |  |  |  |  |
| *tet(W)* | AAGCGGCAGTCACTTCCTTCC | TCAAGTATCCCAGCGAAACC | 60 | 1239 | Tetracyclines | ^23^ |
| *tet(M)* | ACAGAAAGCTTATTATATAAC | TGGCGTGTCTATGATGTTCAC | 55 | 171 | Tetracyclines | ^23^ |
| *tet(O)* | ACGGARAGTTTATTGTATACC | TGGCGTATCTATAATGTTGAC | 60 | 171 | Tetracyclines | ^23^ |
| *tetA(B)* | TTGGTTAGGGGCAAGTTTTG | GTAATGGGCCAATAACACCG | 55 | 659 | Tetracyclines | ^23^ |
| *blatem* | TTTCGTGTCGCCCTTATTCC | CCGGCTCCAGATTTATCAGC | 60 | 690 | Penicilins | ^23^ |
| *blaCTX-M* | ATGTGCAGYACCAGTAARGTKATGGC | GGGTRAARTARGTSACCAGAAYSAGCGG | 60 | 592 | Penicilins | ^23^ |
| *blaSHV* | CACTCAAGGATGTATTGTG | TTAGCGTTGCCAGTGCTCG | 58 | 885 | Penicilins | ^23^ |
| *mecA* | GGGATCATAGCGTCATTATTC | AACGATTGTGACACGATAGCC | 56 | 527 | Penicilins | ^23^ |
| *aac(6")-le-aph(2")* | CCAAGAGCAATAAGGGCATACC | CACACTATCATAACCATCACCG | 55 | 222 | Aminoglycosides | ^23^ |
| *strA* | CTTGGTGATAACGGCAATTC | CCAATCGCAGATAGAAGGC | 65 | 548 | Aminoglycosides | ^23^ |
| *cmlA1* | CACCAATCATGACCAAG | GGCATCACTCGGCATGGACATG | 60 | 115 | Chloramphenicol | ^23^ |
| *blaCMY* | GATTCCTTGGACTCTTCAG | TAAAACCAGGTTCCCAGATAGC | 55 | 1807 | Penicilins | ^44^ |
| *qnr(A)* | ATTTCTCACGCCAGGATTTG | GATCGGCAAAGGTTAGGTCA | 55 | 516 | Quinolones | ^45^ |
| *vanA* | GGGAAAACGACAATTGC | GTACAATGCGGCCGTTA | 55 | 732 | Vancomycin | ^46^ |
| *vanB* | ACCTACCCTGTCTTTGTGAA | AATGTCTGCTGGAACGATA | 55 | 300 | Vancomycin | ^46^ |

***Supplementary figure 1: Temporal development of microbiome composition.***

(A) Relative abundance of bacterial taxa on genus level in stool samples from the first days of life, after 1 month and one year, genera are listed in the order of the most prevalent ones on the top of the legend. (B) Beta diversity measurements via principal coordinates analysis depicted the presence of four distinct clusters representing each of four sample types. (C-E) Comparison of alpha-diversity measurements. Significance was estimated using pairwise Wilcoxon rank sum test (**P* < 0.05, *** *P* < 0.001).


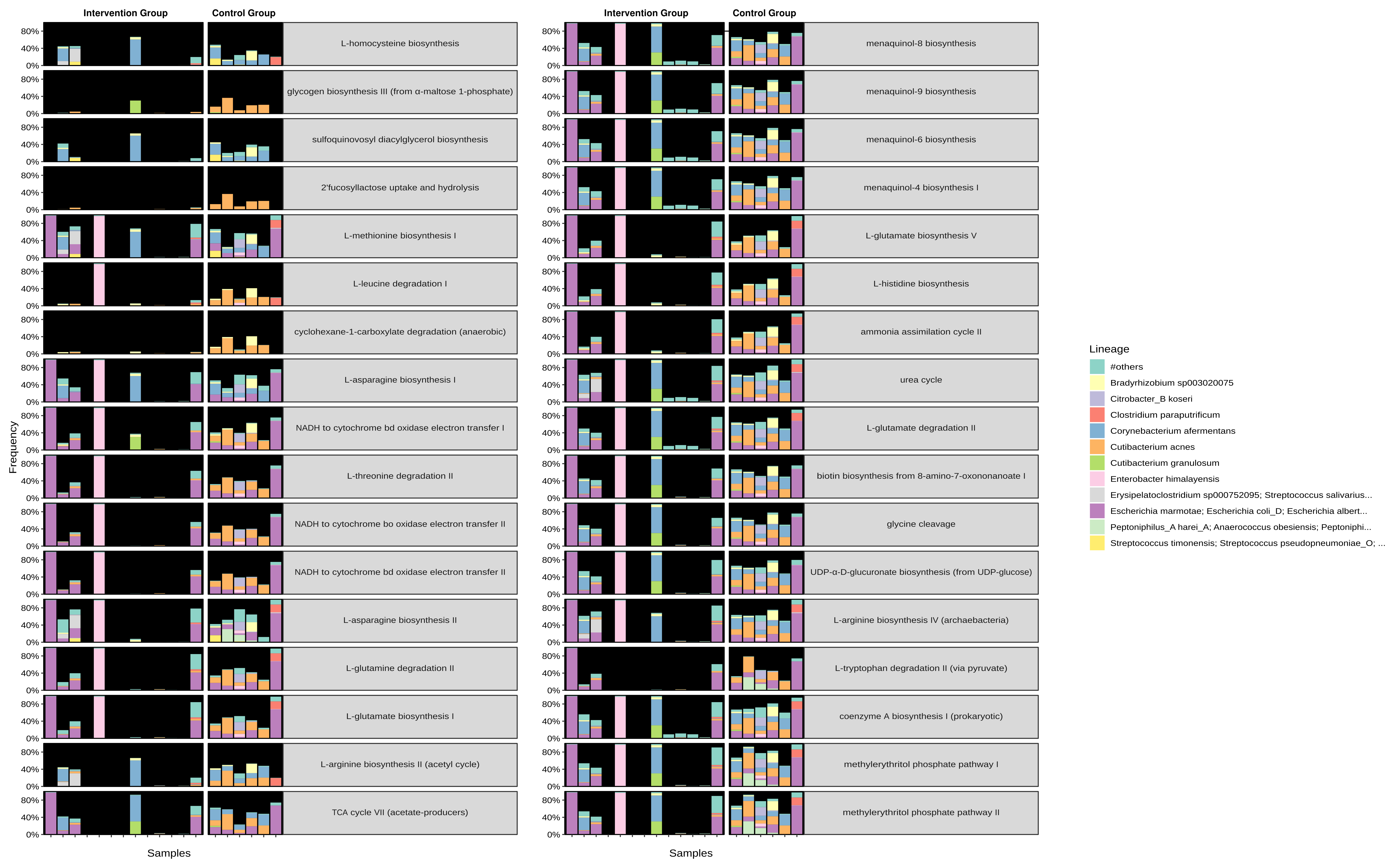
***Supplementary Figure 2: Summed relative abundance of OTUs that were predicted to harbor the metabolic pathway listed in Figure 3 (Day 1).***

Individual abundances are colored based on the taxonomy of the genome from the UHGG which were mapped to the representative OTU sequences from this study.


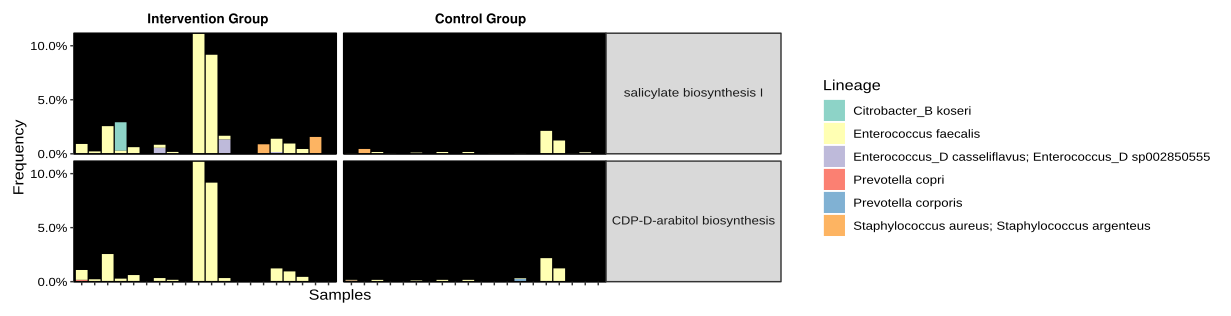
***Supplementary Figure 3: Summed relative abundance of OTUs that were predicted to harbor the metabolic pathway listed in Figure 3 (one month).***

Individual abundances are colored based on the taxonomy of the genome from the UHGG which were mapped to the representative OTU sequences from this study.

***Supplementary figure 4: Comparison of the beta diversity of the microbiome between control and intervention groups at the later timepoints.***

Beta diversity measurements via principal coordinate analysis at 1 month of life timepoint (A) and 1 year (B) depict higher similarity between the intervention and control groups compared to the first days of life, however slight dissimilarities could still be observed.
